# Quackery

**Published:** 1906-02

**Authors:** Dudley F. Sicher

**Affiliations:** Yale University


### TEXAS MEDICAL JOURNAL.

ESTABLISHED JULY, 1885.

PUBLISHED MONTHLY.—SUBSCRIPTION $1.00 A YEAR.

VOL. XXI. AUSTIN, FEBRUARY, 1906. No. 8.

The pnblisher is not responsible for the views of contributors.

### Quackery.

BY DUDLEY F. SICHER, YALE UNIVERSITY.

What impresses one in reviewing the literature, is the extent and ancient origin of quackery, and the ineffectual fight against it. Eight pages of the "Index Catalogue of the Library of the Surgeon General's Office" are taken up with a bare list of books, pamphlets and addresses, exploiting quackery or aiming at last to deal it the long-evaded death-blow. As early as 1605 we find good Dr. Guybon riding out to the charge with "Beware of Pickpockets. Or a Caveat to sick folkes to take heede of unlearned phisitions and unskilfull chyrurgians"; but neither this heavy artillery nor the unbroken fire of subsequent English doctors could daunt the brave hosts of mountebanks who have marched on through the decades, healing the well and making the sick remember their pains. English sovereigns down through Queen Anne continued to exercise the "Royal Touch"; in 1665 one Valentine Greatrakes successfully laid claim to this healing power; a certain Dr. John Ward gloriously humbugged King George the Second; somewhat later, Elisha Perkins (1741-1799), of Norwich, Conn., son and father of Yale graduates, enthralled two continents — laity and physicians alike—with his Metallic Tractors. Then, in the early nineteenth century, *floruit* (on pickings estimated at fifteen thousand pounds per annum) suave John St. John Long, of whom Dr. Francis R. Packard says: "The list of his patients reads like a directory to the fashionable quarter of London." These are only a few, the more gigantic, vermin from out the dirty swarm. Everywhere and everywhen we meet with exploiters of secret remedies, unfailing panaceas, advanced treatment (*sic*), and all other alleged cures which stand as quackery (in the words of Dr. A. T. Schofield) "when used by unqualified men, or if they are advertised or puffed unprofessionally, or connected with any fraud or wilful exaggeration." But it was left for the modern era to furnish that strangest chapter—of an enormous spread of quackery, along with progress in scientific medicine and the growth of education. Berlin, capital city of the world's least hysterical people, reports an increase of 1600 per cent in the number of resident quacks since 1874. For England the roll-call is answered by *The British Medical Journal* thus: "John Bull, for all his boasted common sense and hatred of humbug, is still more quack-ridden than any member of the human family except his cute Cousin Jonathan." And as for "cute Cousin Jonathan's" America—Champe S. Andrews, counsel exclusively retained by the Medical Society of the County of New York to expose medical frauds, is authority for the estimate that in New York City alone there are, against six thousand regular practitioners, twenty thousand quacks. In view, therefore, of its ancient origin, persistence and recent spread, it is not enough to account for quackery on the basis of the Irishman's observation that "there were always fools in this world; in fact, there must have been some lying around, waiting for the world to begin." * * * Rather is quackery a well-defined phenomenon, grounded on effective causes. Why it should exist at all, how the worst empiric enjoys custom, often from the cultured, and what measures may be aimed against this social evil are questions which invite examination.

At the very bottom lies the insufficiency of orthodox medicine. Not even the long strides of the last century have brought it to the full rank of an exact science. The doctor must stand by, and, only half intelligently, assist *vis medicatrix naturæ;* until quite recently at least, he could in no wise control her, like the chemist and the engineer. Rather has he been somewhat in the position of the philosopher, who must work, more or less, in the mist, and between uncertain boundaries. That explains not only the early rites of the medicine-man, but the whole belief in proffered panaceas. The alchemist sought the *one* agent which should turn all the baser metals into gold; the philosopher still seeks the *one* truth which shall uncover heaven's mysteries. Is it not equally natural that men should lend a credulous ear to every announcer of the much-sought cure-all?

Then, to this prospect of a universal medicine we must add the call of the new—always so strong in unsettled provinces. I mean, that something in a wide-awake community or a growing sphere of knowledge which sees salvation in the novel. We recognize this tendency in the fad-worship of Indian occultists, in the rapid succession of new systems of philosophy, in the passing dominance of scientific theories and in the brief vogue of methods in therapeutics. Out of this same phenomenon grows the ready acceptance of Quack A's "Absolutely New Method of Treatment. No Drugs. No Knife" or Empiric B's "Radical Invention. All Diseases banished without Fail."

Remember, moreover, the omnipresence of disease, its agonies and the common dread of it. With this monster the doctor is asked to triumphantly close, whereas he can only pelt it at a distance. When the suit is lost, it is usually the law, not the lawyer, on which the vials of bitterness are poured; how seldom comes a fatal sickness for whose sad issue some doctor isn't blamed! Consider what large proportion of quack remedies is for cancer and incurable female complaints: "The doctors all gave me up," writes Figment A; "I know you have tried the physicians in vain," blares Humbug Z. It is here, upon affections which scientific medicine confesses it can not help, and also upon maladies born of shrewd playing on one's fear of disease, that the empiric waxes fat. Why shouldn't the invalid take heart and believe? Often the loud assurances act as anodyne; occasionally, they even effect a cure. Or, how can the neuropath and the valetudinarian escape the hypnotism of the quack's terrorizing? For the quack wields a deadly weapon in what psychiatrists recognize as "the power of the unconscious mind over the body." He forces credence by calculated emphasis and careful insinuation. He works you into a mood where the mind "autosuggests," at times the throwing off of a disease, more usually, belief in a cure or the assumption of imaginary sickness. It is, of course, a familiar fact that the typical medical student goes through the whole calendar of diesases. "Autosuggestion" is the technical word for this mysterious process; it is what the hypnotist employs, but never to stronger purpose than the superior quack.

Given, on the one hand, this set of causes—the limitations of scientific medicine, the pain and dread of disease, and the power of "autosuggestion," and, on the other hand, depraved humanity, hard-driven in the struggle for existence, but cunning in the knowledge of men, and you have the essential parts which, with a few minor pieces, make up into the smooth engine of quackery.

Every newspaper and magazine reader knows how well the quack makes capital out of the limitations of scientific medicine. When the regular practitioner is puzzled, he admits, or when the case transcends cure, he gravely shakes his head. The quack now steps in and begins where the other left off. He "especially solicits obstinate cases"; "welcomes the doubter and the skeptic." He realizes the persuasive value of bold assertion and big promises; how the exclamation-point and the period may appeal more strongly than the careful interrogations of the honest physician. He talks much of the "thousands who testify to its success," and thus swaggers himself into the confidence of the poor invalid, whom the doctors, in good conscience, must acknowledge beyond their aid. With so many broken-hearted witnesses of the insufficiency of evolved therapeutics, almost any knave can steal a living by brazenly opposing some dominant practice in medicine—as surgery or the use of drugs. These "methods" nowadays have a pseudo-physiological basis; with a speciousness it is often hard to confute, tracing all disease back to "inside nerves," "sluggish circulation," and the like, they impress by the sweep of their assertion and their tone of scientific explanation. It is scant wonder that the pompous logic moves the incurable, whom neither "knife" nor "drugs" can save, vapid ladies of fashion, and the smart shallowpate of "a little learning."

But the quack does not depend solely on the agony of disease and the inability of scientific medicine completely to cope with it. He swells the total of victims by magnifying minor ailments and imposing imaginary ones. By cooked mortality statistics he frightens the individual into noticing and treating some indisposition, which the family doctor, generally to no effect, laughingly pronounces not worth bothering about; and then, conversely, by the same process of "autosuggestion," a few months' trustful application of the vaunted nostrum brings back the patient's assurance and draws his mind from the ailment. Or, the quack will address himself to the social weakling, and by skilful insistence ascribe failure to "pelvic disease," "nerve exhaustion," "*and all that*," as Pope says. The poor numskull and the unattractive girl are quick to seize the hope; yes, not deficient endowments, but dissipation or insidious disease has caused their defeat—good Doctor Slyfox, A. B., M. D., member of six medical institutes and nineteen learned societies, will raise them out of the slough. Again, along this same line of "autosuggestion," the quack enlarges his levée by invitations to self-diagnosis. With a subtle mastery of rhetoric he sets forth such an array of "symptoms" that no diligent pupil need feel he is cast into outer darkness. Follow the fraudulent guide—and yesterday you had consumption; today varicocele fastens you in its fangs; tomorrow your kidneys will be fatally weak—and so the falsehood runs.

It may be supposed that caution so palpably absurd would rouse more ridicule than credence. But the hypochondriac, the neuropath, the person of weak judgment (ignorance is no indispensable factor) do not reason in such matters. We are almost led to accept as genuine the testimonial in which it is written, "I had tried *all* the medicines." With such people, the high-sounding swagger, pretended altruism and adroit description of past achievements drown out the voices of common sense. Even the normal reader can hardly turn to the quack's advertisement day after day, in a non-critical mood, without experiencing at least a passing influence. The fulsome notices of books and plays, in fact, the whole psychology of advertising, rest on this very principle of "autosuggestion." So all the quack requires is a hearing. Given a hook-and-line and a pond of fish, he understands baiting too well, not to land a heavy catch.

Of course, there are contributory factors. The quack has other resources. Notable is his use of that universal weakness, the basis of get-rich-quick schemes and the shopper's bargain,—I mean the fascination of getting something for nothing. The doctor will send you a heavy bill on the first of January or July; the quack offers: "No Pay till Cured," "Send for Sample Bottle. Free." How the charlatan manages never to lose out would make a realistic novel in itself. Suffice it to indicate his crafty reliance on creating "the habit"; one bottle with its high content of alcohol will inevitably "tone you up," or admixed opiates may be the "irresistible pain-killer," to which you will want to turn again. Quacks are among the largest customers of wineries and distilleries. Recent analyses (by the Massachusetts State Board of Inquiry) have developed the possibility that the druggist's show-case may hold more alcohol than the cellar of the saloon opposite, and many a temperance advo cate, quite unknowingly, has drawn inspiration for his lecture from that after-dinner glass of nerve tonic or stomach bitters.

With such instruments at his disposal, restrained by no Hippocration oath or sacred reputation, left to run riot, by criminally lax laws, deliberately dead-lettered, the *genus* Quack swarms out over the land. Its species are unnumbered, being marked by every device deceitful ingenuity can conceive. Psycho-therapeutics and knowledge of human nature constitute the quack's entire outfit; all he really needs is moral atrophy and the instincts of a cheap drummer. Such is the baleful etiology of medical quackery.

If confirmation of this diagnosis is desired, it may be sought in the recent spread of quackery and its especial vogue in America. Paradoxical as it sounds, the growth of education, while compelling the quack to improve his methods, has greatly extended his field. Formerly, he seldom worked farther than his voice or circular might carry; now, every literate is a potential victim. His wares are displayed in almost every piece of print that strikes your eye; for the publisher and "the press" he has subsidized and suborned. So-called family magazines (messes of popular fiction and indecent advertisements) are distributed gratis at the instance and backing of the quack, for whom they are so much purchased propaganda. To the same end he sustains the whole modern plethora of magazines and newspapers. Without his lucrative patronage periodicals, representing the real excess of supply over demand, would end their artificial existence, and so, wherever there is a struggling paper, manhood slumbers and the editor accepts the proffered bribe. How else explain the significant truth that the sectarian press ranks among the worst offenders? The "yellies," too, depend as much upon the quack as upon scandal; and the most prosperous of them all affords the grossest example. The editorial columns of a certain evening journal will, no doubt, tonight, blare its owner's championship of the people, while almost every page invites the trust-ground toiler to hand up his savings to swindling men specialists and venders of alcoholic cure-alls. In fact, with a few notable exceptions, such as *The Outlook*, *Life*, and *The New York Evening Post*, the whole press unblushingly sprinkles its columns with the charlatan's cards. Nearly every New York daily on January 24 reported, at lengths inversely proportioned to its abetment of quackery, the exposé of Dr. Henry Kane's Radium-Cure swindle; in the January 22 issue of one of the most reputable of them, I find a conspicuous advertisement of this same discomfited wonder-worker. Shameless self-interest never could have played so slavishly into the quack's hands until the growth of education made publishing a fiercely competitive business.

At the same time, not only has the growth of education placed a megaphone to the empiric's lips, but it has sensitized the public to his call. There is a wider interest in hygiene and therapeutics; people think more about their health and more readily take alarm. "Health journals" enjoy large circulations; too often nothing more than elaborate handbills of the editor's particular book, "system,"

or hygienic contrivance, and, at best, running wild with "hints to health" and philippics against the doctors, these magazines only succeed in leaving their readers the shuttlecock of every battledore in quackdom. Similarly, the broadcast discussion of medical problems, in response to the interests of an educated public, creates a kind of diathesis to imaginary disease. Then, vaguely bound up, perhaps, in widespread education is the modern stress of life, high nervous tension and susceptibility to fads.

As a final (undetached) cause we must recognize the passion for untrammeled personal freedom, so characteristic of latter days, especially in England and America. It is that attitude which one writer savagely describes as "jealousy safeguarding to every citizen the sacred right of going to the devil in his own way." Fearing to dispense undue privileges and unjust fetters, framers and executors of the law, notably in the United States, have virtually thrown open the delicate art of healing to almost any person too crack-brained or dishonest to earn an honorable living. Not only does quackery thus recruit directly, but wild-cat schools are permitted to dump upon an overcrowded profession graduates sadly lacking in capacity and training. Most of these must end up as charlatans, in much the same way as the manufacturer, shut out from the restricted trade in the genuine article, caters to the public taste with cheap and tinsel imitations; or, at best, such half-baked doctors impair the efficiency of their brotherhood and shake confidence in it. It is not bare accident that America is at once the "home of quackery" and the "home of the free."

These, therefore—growth of education and the modern spirit of liberty—are the specific forces behind the recent spread of quackery; and America stands as arch-victim, just because they have been at their strongest here.

Even from the foregoing generalizations it must appear that quackery is a seated evil, which the community, in self-defense, ought promptly to weed out. Yet the roots, as we have seen, spread out so variously, that past effort has been without effect, and the future will do no better unless exceptional measures are applied. In this case, it seems, diagnosis is easier than treatment, for the social physician is blocked on every side. Surely, the requirements should be everywhere approximately as high as the better States and countries have set, yet every step towards restriction of practice, even to the safety-point, meets with wrangling opposition. The cry of paternalism is raised, and even the disinterested see in such measures only an attempt at extending the alleged "Medical Trust."

Quarantine is proper; government exposure of food adulteration is only right; of course, the State should protect its citizens against fraudulent investment schemes, and every enforcement of these safeguards calls out general praise. But it is ruinous paternalism to save the unwary public from unconscious alcoholism, medical extortion and dangerous malpractice!

Of the same caliber, it seems to me, is that other plea against State interference, to the effect that variance from orthodox practice is not enough to brand a method as quackery. It is urged that progress consists in dissidence, and that the traditional school has no right to sit in judgment. "The prophet is never believed in his own country," you know. Such argument—and it is very common—sounds too much like the prattle of those "advanced thinkers" who would do away with the moral code on the ground that all standards are relative and arbitrary. Further, the records fail to show a single instance where scientific medicine has drawn profit from quackery, nor is the modern broad and progressive attitude likely to cheat any honest radical of an adequate hearing.

Just so long, however, as this repugnance for State interference with medical quackery obtains, it is folly to seek help in that quarter. *Existing* postal laws and statutes on fraud are themselves sufficient to blackeye quackery, and their total failure stands as a pathetic proof of the scant likelihood of ending quackery through the toils of the law. Mr. Andrews reports that a wellnigh insuperable obstacle to his vigorous work is the difficulty of obtaining witnesses; persons are rather diffident about exposing frauds of which they have been the stupid victims. Besides, even in clear cases of fraud, it is often impossible to lay hands on the real culprit; or, if caught, after paying his fine or serving his sentence, the quack can start up the old business in another section under another name; the salutary restraints of public opinion play no part with him. After all, what boots it to crush a dozen or even fifty out of the unnumbered swarm? The press will not emphasize the prosecutions, and so their effect is lost. Similarly, the postal department could, quite legally, I believe, stamp out the evil alone, if it dared exclude from the mails every periodical containing a single fraudulent quack advertisement; but how prove its case, and where is the administration which could survive the ensuing clatter about "usurpation of authority" and "freedom of the press"?

Legislation, therefore, can only be secondary to ventilation and the education of public opinion. But how educate public opinion, when its educator, the press, is itself irretrievably allied with the forces of evil?

First, obviously, such papers as have not prostituted themselves must agitate; they should expose their brothers' shame and the people's consequent losses. Editor Bok's recent appeal (in *The Ladies' Home Journal*) to the women of the land not to let their babies suck in with their milk the alcohol or opium of "motherhood" nostrums, and to tear down from fence and barn the quack's advertisement, is the kind of measure that counts. Here, too, is a chance for those wealthy yellow journals, forever bruiting their own altruism, to whom a "scoop" is more necessary than the quack's gold, to expose typical quacks; they make easier handling than the gas and the beef trust, and the attack, no doubt, would yield even richer sensations than the divorce court. Then, public-spirited men of all professions should everywhere organize — as has just been done in Germany—a systematic campaign against quackery. The recent example of an English workingman's society should be followed, and illuminating tracts be circulated by unions and employers. Perhaps the school boards may be free also to level a blow. I know the tendency is to overcram the curriculum, to attempt to arm the child with a pretty smatter against every need in life; but if we are going to teach hygiene at all, if the possible consequences of alcohol and tobacco are to be pointed out, why not lay some stress on a curse just as extensive and no less harmful, one which rests on no natural appetite, but on ignorance and absence of forewarning? At any rate, superintendents of boards of free lectures can include in their admirable courses a few talks on quackery by such qualified experts as Champe S. Andrews, Esq.

Against measures of this sort the press hardly dares raise its voice, and effective legislation will soon follow as the expression of the popular will.

Such procedure, it is hoped, may limit the future annals of quackery, and hasten that golden age when even the doctors can almost agree with Mrs. Eddy that there is no such matter as disease.—*Popular Science Monthly.*

